# A Review of Antimicrobial Activity of Dental Mesenchymal Stromal Cells: Is There Any Potential?

**DOI:** 10.3389/froh.2021.832976

**Published:** 2022-01-14

**Authors:** Oleh Andrukhov, Alice Blufstein, Christian Behm

**Affiliations:** ^1^Competence Center for Periodontal Research, University Clinic of Dentistry, Medical University of Vienna, Vienna, Austria; ^2^Division of Conservative Dentistry and Periodontology, University Clinic of Dentistry, Medical University of Vienna, Vienna, Austria

**Keywords:** dental mesenchymal stromal cells, antimicrobial peptides, oral health, immunomodulation, Vitamin D_3_

## Abstract

Antimicrobial defense is an essential component of host-microbial homeostasis and contributes substantially to oral health maintenance. Dental mesenchymal stromal cells (MSCs) possess multilineage differentiation potential, immunomodulatory properties and play an important role in various processes like regeneration and disease progression. Recent studies show that dental MSCs might also be involved in antibacterial defense. This occurs by producing antimicrobial peptides or attracting professional phagocytic immune cells and modulating their activity. The production of antimicrobial peptides and immunomodulatory abilities of dental MSCs are enhanced by an inflammatory environment and influenced by vitamin D_3._ Antimicrobial peptides also have anti-inflammatory effects in dental MSCs and improve their differentiation potential. Augmentation of antibacterial efficiency of dental MSCs could broaden their clinical application in dentistry.

## Introduction

One of the main prerequisites for oral health is the maintenance of the host-microbe homeostasis in the oral cavity [1, 2]. A symbiotic relationship between the oral microbiome and the host immune system is maintained by numerous complex mechanisms and exhibits resilience against various external stimuli [3, 4]. Many oral diseases, including caries and periodontal disease, are associated with disruption of this homeostasis and dysbiosis [5, 6]. Besides the host immune system, the production of antimicrobial peptides (AMPs) is an important factor in controlling the oral microbiome, health maintenance and in developing various oral pathologies [7].

Mesenchymal stromal/stem cells (MSCs) are plastic adherent fibroblast-like cells expressing specific surface markers (expression of mesenchymal markers CD29, CD73, CD90, CD105, while lacking expression of hematopoietic markers CD14, CD34, CD45) and exhibiting multipotent differentiation capacity to osteoblasts, adipocytes, and chondrocytes [8, 9]. Initially, these cells were isolated from bone marrow, but later MSCs were found in various other adult tissues, including almost all dental tissues [10, 11]. Although MSCs are recognized as a powerful tool in regenerative medicine, the mechanisms of their action *in vivo* are rather elusive. Their differentiation potential *in vivo* and survival after the transplantation is limited, and their therapeutic effect is supposed to be mainly achieved by secretion of trophic factors and immunomodulation [12–14].

Recently, a potential contribution of MSCs into antimicrobial defense was reviewed. Two major possibilities for antimicrobial activity were proposed, namely a direct and an indirect mechanisms [15]. Directly, MSCs may produce various antimicrobial products, like AMPs, interleukin (IL-)17 and indoleamine-2,3,-dioxygenase (IDO). Indirect antimicrobial effects of MSCs are associated with the modulation of the phagocytic activity and the production of numerous chemoattractants stimulating their recruitment. However, MSC's input into antimicrobial defense remains controversial and rather limited. Even less is known on the antimicrobial activity of dental MSCs. Therefore, the present review attempts to summarize and critically discuss state of knowledge about this topic. As in our previous reviews [14, 16], information from studies with confirmed MSC's phenotype as well as from those using fibroblasts-like cells will be included.

## Antimicrobial Activities of Dental MSCS

### Antimicrobial Peptides

Antimicrobial peptides are small natural peptides produced by multicellular organisms and possess antibacterial activity [17]. Most AMPs are positively charged and therefore can easily bind negatively charged bacteria and kill them by incorporation in their cytoplasmic membrane [18]. As recently reviewed, human AMPs exhibit a cytotoxic activity toward numerous oral bacterial species in planktonic and biofilm forms [19]. Three major types of oral AMPs are reported in humans: defensins, cathelicidins, and histatins [20].

Defensins and cathelicidin are the only AMPs reported to be produced by dental MSCs. Defensins in humans are subdivided into two large subfamilies: α-defensins (human neutrophil peptides, HNP) and β-defensins (hBDs), which further comprise several peptides encoded by various genes. HNPs are mainly produced by neutrophils, while hBDs are produced by epithelial cells [21, 22]. Human defensins exhibit antibacterial activity toward numerous oral species, including *S. mutans, E. faecalis, A. naeslundi* and *Lactobacillus spp*. [23, 24]. The only cathelicidin described in humans is LL-37, which is mainly produced by neutrophils, monocytes, and lymphocytes and inhibits growth and biofilm formation by various oral species [25, 26].

### Defensins

Earlier studies denied the expression of hBDs in human gingival fibroblasts (hGFs), which can be explained by the methodological limitations at that time. Krisanaprakornkit et al. [27] showed using reverse transcription PCR that the hBD1 gene is not expressed in hGFs, in contrast to gingival epithelial cells. Another study using reverse transcription PCR did not detect the expression of hBD1, hBD2, and hBD3 in hGFs [28]. However, later studies in hGFs using more sensitive detection methods implied that these cells produced different hBDs. Most studies suggest that the expression of defensins in dental MSCs is regulated by various inflammatory stimuli. Rizzo et al. [29] revealed that the expression of hBD2 in hGFs is activated by viable but not heat-inactivated *Chlamydia pneumoniae*. Dommisch et al. [30] showed that *Porphyromonas gingivalis* and *Streptococcus gordonii* increased mRNA expression of hBD2 and hBD3, but not that of hBD1 in hGFs. IL-1β upregulated the expression of hBD1 and hBD2 and downregulated that of hBD3 in hGFs [31]. Jang et al. [32] demonstrated that the expression of hBDs in hGFs is also regulated by various oral bacteria in a different way: the expression of hBD2 is stimulated by *Leptotrichia wadei*, whereas the expression of hBD3 is inhibited by this species as well as by *P. gingivalis*. Besides direct inflammatory stimuli, the expression of hBD2 in hGFs was increased in the wound healing model after scratching the cell monolayer [33].

In periodontal ligament-derived MSCs (hPDLSCs), the expression of hBDs was shown to be regulated not only by inflammatory stimuli but also by vitamin D_3_. Vitamin D_3_ is a steroid hormone involved in the regulation of bone metabolism and immune response [34, 35]. The major form of vitamin D_3_ in the blood is 25(OH)D_3_, which is further converted into biologically active 1,25(OH)_2_D_3_ in the kidney [36]. There is some evidence that the bioactivation of vitamin D_3_ might also occur in the oral tissues [37]. De Filippis et al. [38] showed that the production of hBD3 in human periodontal ligament cells (hPDLCs) is stimulated by *P. gingivalis* lipopolysaccharide (LPS) and vitamin D_3_. Moreover, hPDLSCs-derived hBDs inhibited *P. gingivalis* growth [38]. hPDLCs also express defensin alpha 4, which is upregulated by LPS and regulated by epigenetic mechanisms [39, 40].

A study on odontoblast-like cells showed that these cells express both hBD1 and hBD2 [41]. Interestingly, the recombinant hBD2 decreased hBD1 expression, did not influence hBD2 expression and increased the production of various inflammatory cytokines by these cells [41]. Production of hBDs in dental pulp-derived MSCs is also increased by inflammatory stimuli. Thus, hBD2 gene expression in human dental pulp cells (hDPCs) was upregulated by LPS [42] and muramyl dipeptide, a cell wall component of both Gram-positive and Gram-negative bacteria [43]. Another study showed that the gene expression and protein production of hBD2 in hDPCs is increased by tumor necrosis factor (TNF)-α and IL-1β in a synergistic manner [44].

### Cathelicidins/LL-37

Compared to defensins, there is less evidence on the production of LL-37 by dental MSCs. To date, there are only two reports showing that gene expression and secretion of LL-37 in hGFs and hPDLCs was substantially upregulated by 25(OH)D_3_ and 1,25(OH)_2_D_3_, which was further enhanced by *P. gingivalis* lipopolysaccharide [45, 46].

### Effects of AMPs on the Inflammatory Response and Regenerative Potential of Dental MSCs

Besides antimicrobial function, AMPs are also known to influence the characteristics of dental MSCs. As recently reviewed, LL-37 exerts an anti-inflammatory effect in hPDLCs [47]. In hGFs, LL-37 exerted not only an anti-inflammatory effect but also stimulated the production of various growth factors involved in tissue remodeling [48]. hBD3 and LL-37 exhibit synergistic anti-inflammatory effects in 3D co-culture models of gingival epithelial cells and fibroblasts [31]. In contrast, one study suggested that the effect of LL-37 in hGFs may be either anti-inflammatory or pro-inflammatory depending on the present Toll-like receptor (TLR) agonist [49]. In addition, hBD3 inhibited the inflammatory response in hDPCs [50]. AMPs might also influence the differentiation capacity of dental MSCs. Recent reports showed that hBD3 enhanced osteogenic differentiation of hPDLCs [51], whereas LL-37 enhanced odontogenic differentiation of hDPCs [50].

### Indoleamine 2,3 Dioxygenase

The major function of IDO is the depletion of tryptophan by its conversion into L-kynurenine, which results in the inhibition of the immune response. Besides dampening the immune response, tryptophan depletion also inhibits bacterial growth; moreover, it happens even earlier than immunosuppression [52]. Human MSCs stimulated with different cytokines exhibited IDO-mediated antibacterial, antiprotozoal, and antiviral effects *in vitro* [53]. Interestingly, IDO-dependent antimicrobial effects were not observed in murine MSCs, which do not express IDO. Instead, murine MSCs might inhibit bacterial growth through nitric oxide production by inducible nitric oxide synthase [53].

The expression of IDO in resting dental MSCs is low and is stimulated by various inflammatory cytokines such as TNF-α, IL-1β, and interferon (IFN)-γ, and to a lesser extent by TLR agonists, mainly by TLR3 activation [14, 16, 54]. The highest IDO expression and activity in dental MSCs is achieved upon the stimulation with IFN-γ [55, 56]. However, the exact role of MSC-derived IDO in bacterial defense has to be further investigated.

### Indirect Modulation of Antimicrobial Activity by Dental MSCs

Similar to MSCs from bone marrow and other tissues, dental MSCs exhibit immunomodulatory properties and can influence the activity of almost all types of immune cells [14, 57, 58]. The immunomodulatory ability of dental MSCs is strongly upregulated by various inflammatory cytokines and some Toll-like receptors [14, 58]. Among others, dental MSCs regulate the activity of polymorphonuclear neutrophils (PMNs) and macrophages, the cells of the innate immune system primarily involved in infection control. Additionally, dental MSCs produce high amounts of various chemoattractants and thus stimulate bacterial killing via recruitments of phagocytic immune cells.

PMNs, the most abundant type of white blood cells, continuously migrate in the gingival sulcus and play an essential role in controlling oral infection and biofilm growth. PMNs phagocytose the invading bacteria and kill them by releasing various antibacterial enzymes or producing reactive oxygen species (ROS). A further antimicrobial effect of PMNs is the release of neutrophils extracellular traps (NET) after their death [59]. Few studies showed that the antimicrobial functions of PMNs could be influenced by dental MSCs. Blufstein et al. [60] showed that conditioned media of IL-1β-treated gingival MSCs have an anti-apoptotic effect on PMNs isolated from peripheral blood. This was not observed for untreated or TNF-α-treated cells. No effect of gingival MSC's conditioned media on ROS production by PMNs was observed. An extended lifespan of PMNs could mean that their ability to phagocytose invading pathogens remains for a longer time and could be interpreted as enhancement of antimicrobial activity. Misawa et al. [61] investigated the effect of conditioned media of differently treated hPDLSCs on the functional activity of human promyelocytic leukemia HL-60 cells sharing many properties of PMNs. Phorbol myristate acetate-induced ROS production by HL-60 cells was enhanced by the supernatants of hPDLSCs treated by *P. gingivalis* protein extract and decreased by those of untreated hPDLSCs. Thus, it seems that dental MSCs might potentially improve the antimicrobial function of PMNs, especially in the inflammatory environment.

Macrophages are professional phagocytic cells of the innate immune system and, besides bacteria recognition, are involved in numerous processes such as the clearance of apoptotic cells, regulation of tissue repair, and homeostasis [62, 63]. The macrophages exhibit high plasticity and their function is determined by environmental factors [64, 65]. In a very simplified view, macrophages are polarized either into pro-inflammatory M1 phenotype or anti-inflammatory M2 phenotype [65]. Both types of macrophages possess a strong phagocytic ability compared to non-primed M0 macrophages [66].

As previously reviewed, in most cases, dental MSCs shift the polarization of macrophages toward the M2 phenotype [14]. It is not entirely clear how such polarization will influence the antibacterial activity of macrophages. On the one hand, pro-inflammatory M1 macrophages drive the immune response and promote bacterial clearance [67]. On the other hand, there is evidence that M2 macrophages can exhibit an even higher phagocytic activity and ROS production than M1 phenotype [68].

It should also be noted that most of the studies showing the shift of macrophages toward M2 phenotype in the presence of MSCs performed their experiments with macrophages derived from monocytic cell lines or even mouse macrophages. The situation in primary human macrophages could be less obvious. Our study showed that the conditioned media of differently primed macrophages can stimulate or inhibit the expression of both pro- and anti-inflammatory markers in primary human macrophages [69].

Tzach-Nahman et al. [70] investigated the effect of conditioned media of human periodontal ligament fibroblasts and hGFs on macrophages derived from a monocytic THP-1 cell line. Conditioned media of dental fibroblasts decreased the production of TNF-α and enhanced macrophage phagocytosis of *P. gingivalis*. These effects were promoted in fibroblasts primed with inflammatory stimuli or derived from inflamed peri-implant tissue. However, it should be considered that *P. gingivalis* might survive phagocytosis by M2 macrophages [71].

Besides direct immunomodulatory effects, dental MSCs produce a high amount of chemoattractants upon stimulation with bacterial products or inflammatory cytokines [16, 72, 73]. The main chemoattractants produced by dental MSCs are IL-8 and monocyte chemoattractant protein 1 (MCP-1), which promote migration of phagocytic PMNs and monocytes, respectively, [74, 75], and thus enhance bacterial elimination. Besides, a recent study showed that MCP-1 produced by equine MSCs stimulates the production of AMPs by primary keratinocytes isolated from horse skin [76]. The existence of such mechanisms for dental MSCs and oral epithelial cells should be considered by further studies.

## Conclusion and Future Perspectives

Host antimicrobial peptides are considered as a prospective tool in the treatment of diseases associated with polymicrobial biofilms [77] and particularly periodontal disease and endodontic infections [78, 79]. Dental MSCs exhibit some antimicrobial properties through the production of AMPs and immunomodulation. The contribution of these mechanisms to antibacterial defense is not yet clear. However, stimulation of the endogenous production of AMPs could be a good strategy for oral health maintenance. This could be achieved, for example, by increasing systemic and/or local levels of vitamin D_3_. Another possibility to increase AMPs production by dental MSCs is their transfection with specific genes. For example, transfection of hPDLSCs or sheets of hPDLSCs with hBD3 have anti-inflammatory and antibacterial effects toward different periodontal pathogens [80].

The occurrence of antibiotic resistance increased within the last decades and is one of the biggest challenges in medicine, particularly in dentistry [81–83]. The development of alternative strategies for antimicrobial defense is one of the challenges of contemporary dental research [84]. MSCs antimicrobial activity could be a useful instrument to overcome this problem. Combination of amoxicillin therapy with MSCs pre-activated with TLR3 agonist Poly I:C exhibited a synergistic antimicrobial effect *in vivo* [85]. Recently, the secretome of equine MSC was shown to inhibit planktonic growth and biofilm formation of various bacteria, including methicillin-resistant Staphylococcus aureus [86]. Naturally produced AMPs or the secretome of AMPs producing cells could theoretically help to overwhelm the problem of increasing antimicrobial drug resistance.

MSCs are a powerful instrument in regenerative dentistry and immunotherapy. Strategies to enhance their antimicrobial properties are promising to improve their efficiency in clinical applications and particularly dentistry. Transfection of MSCs with AMPs results in antibacterial effects and might also improve regenerative processes [87]. Current studies suggest that the production of AMPs by dental MSCs is regulated by bacterial products, inflammatory cytokines and vitamin D_3_. However, the information on the regulation of AMPs production is still limited and further research in this field is necessary. Understanding the antimicrobial activity of MSCs and their role in health and disease would be very important for improving current therapeutic approaches in dentistry.

## Author Contributions

OA, CB, and AB: conceptualization. OA: writing—original draft preparation. CB and AB: writing—review and editing. All authors have read and agreed to the published version of the manuscript.

## Funding

This research was funded by Austrian Science Fund FWF, projects P 29440 and P 35037 (to OA).

## Conflict of Interest

The authors declare that the research was conducted in the absence of any commercial or financial relationships that could be construed as a potential conflict of interest.

## Publisher's Note

All claims expressed in this article are solely those of the authors and do not necessarily represent those of their affiliated organizations, or those of the publisher, the editors and the reviewers. Any product that may be evaluated in this article, or claim that may be made by its manufacturer, is not guaranteed or endorsed by the publisher.
